# Serum HBV RNA: a promising biomarker for blood product safety screening and enhanced diagnostic efficiency in chronic hepatitis B virus infection

**DOI:** 10.3389/fpubh.2023.1248878

**Published:** 2023-08-31

**Authors:** Sulan Yu, Yanjuan Guo, Chunxiao Zhang

**Affiliations:** Central Blood Station of Lianyungang, Lianyungang, China

**Keywords:** blood safety, HBV RNA, biomarker, safety screening, diagnosis

## 1. Introduction

The prevalence of Chronic Hepatitis B Virus (HBV) infection remains a considerable challenge to global health, impacting around 30% of the global populace (1). Although antiviral therapies have made significant progress, the complete elimination of the virus, specifically the covalently closed circular DNA (cccDNA), continues to be a challenging objective. Conventional diagnostic techniques, including HBV DNA quantification and serological markers, possess certain limitations in precisely reflecting viral activity and treatment response (2). Liu et al. reported the identification of serum HBV RNA as a biomarker has demonstrated encouraging prospects in enhancing diagnostic efficacy and screening for blood product safety in individuals with chronic HBV infection (3). The objective of this commentary is to examine the importance of detecting serum HBV RNA, recent advancements in enhancing its diagnostic precision, its potential application as a biomarker for screening blood products for safety, and the consequences for medical practice.

## 2. The significance of serum HBV RNA detection

Blood product safety screening biomarkers refer to distinct molecules or indicators employed to evaluate the quality and safety of blood products that are designated for transfusion or other therapeutic applications. Biomarkers are essential in the identification and mitigation of infectious diseases, including viral infections, bacterial contamination, and other hazardous agents that could be present in donated blood (4). The principal aim of screening blood products for safety is to detect and eradicate any plausible hazards linked to transfusions, thereby ensuring the protection of the recipients against unfavorable responses or transmission of contagious illnesses. The biomarkers utilized in this procedure are commonly assessed via laboratory examinations and function as indicators of diverse infectious agents or other anomalies that could potentially jeopardize the safety of blood products (5).

Serological tests are commonly employed by blood donation centers to screen donated blood for the existence of HBV. The tests are designed to identify particular antibodies against HBV antigens, namely hepatitis B surface antigen (HBsAg) and hepatitis B core antigen (anti-HBc). Blood units that have been donated and test positive for these markers are typically deemed unsuitable for transfusion in order to avoid the transmission of HBV to the recipients (6). Moreover, the term “window period” pertains to the duration between the acquisition of HBV infection and the emergence of discernible antibodies. During this temporal phase, an individual has the potential to contract HBV, however, they may exhibit negative results for HBsAg and anti-HBc upon testing. In order to mitigate the potential for transmission of Hepatitis B Virus (HBV), blood centers frequently employ supplementary measures, including nucleic acid testing (NAT). This method is capable of identifying the genetic material (HBV DNA) of the virus during the window period (7). In some cases, individuals with chronic HBV infection may have very low levels of HBV DNA in their blood, referred to as occult HBV infection. This poses a challenge in detecting HBV using standard serological tests alone. Therefore, in certain situations, blood centers may employ molecular techniques, like polymerase chain reaction (PCR), to quantify HBV DNA levels and assess the risk of transmission more accurately (8). The diagnostic significance of HBV RNA in comparison to HBsAg, anti-HBc, or HBV DNA is not fully established, however, since HBV RNA is still in the clinical research stage and has not yet gained widespread acceptance in clinical practice.

**Figure 1:**
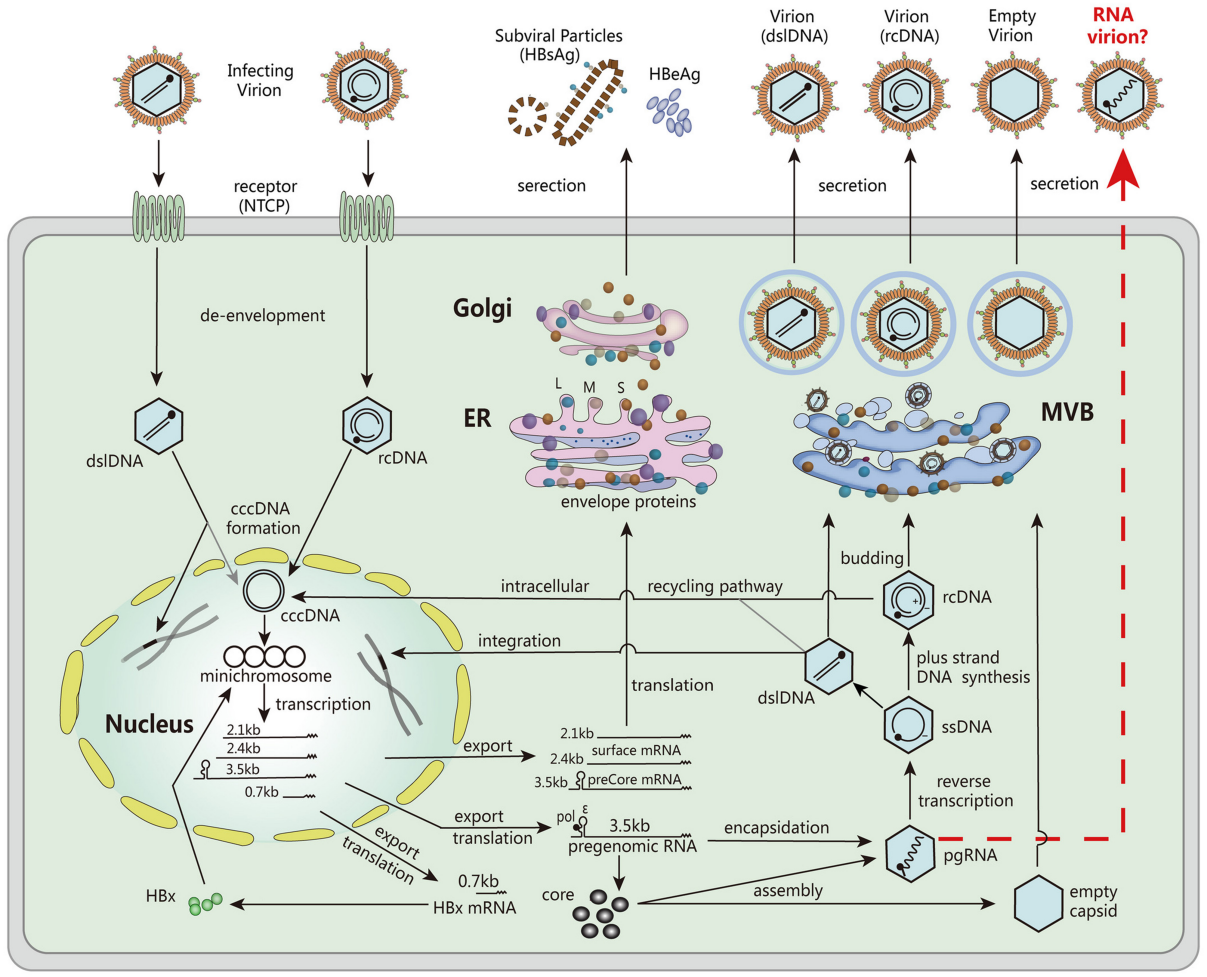
The major steps involved in the HBV life cycle. These steps include viral entry into host cells, de-envelopment of the viral particle, formation of cccDNA, transcription of mRNA, translation of viral proteins, encapsulation of pgRNA, DNA replication, assembly of viral particles, and secretion. Reproduced with permission (3). Copyright 2019, Wiley-VCH.

Serum HBV RNA, specifically non- or partially reverse-transcribed pregenomic RNA (pgRNA) present in HBV virion-like particles, has emerged as a valuable surrogate marker for assessing viral activity and treatment response (9). Unlike HBV DNA, which can be lost during antiviral therapy, serum HBV pgRNA persists and provides valuable insights into the transcriptional activity of cccDNA (Figure 1). Monitoring serum HBV RNA levels has demonstrated its potential to predict treatment efficacy, evaluate the risk of viral rebound after drug withdrawal, and guide clinical decision-making (3, 10, 11).

Recent studies also identified the characteristics of chronic hepatitis B (CHB) participants who are positive HBV RNA. A study by Janssen et al. found that factors associated with serum HBV RNA level were HBsAg status, serum ALT, HBV genotype, and the presence of basal core promoter mutations (12). Ghany et al. reported HBV RNA was also quantifiable in 99% of positive HBsAg donors and 58% of negative HBsAg donors, and HBV RNA level was closely associated with CHB phages, but because of its close relationship with HBV DNA, HBV RNA as a marker also has significant limitations in additional information related to clinical disease indicators, necessitating further verification in large-sample clinical trials (13).

## 3. Optimizing serum HBV RNA detection

According to a study by Yu et al., HBV pgRNA undergoes dynamic changes in splicing variation and truncation at its 3′ terminal in several cohorts of people with chronic HBV infection (14). In this study, reverse transcription-quantitative polymerase chain reactions (RT-qPCRs) were used to target several areas of the HBV genome, and the amount of HBV RNA found in serum was assessed in relation to these RNA species. According to the findings, there is a correlation between which region of the HBV genome is amplified and how precise and sensitive the detection of HBV RNA in serum. The detection levels at the preC/C-RNA region consistently exceeded those at the SF-RNA section and the XR-DNA region. As a result, detection at the preC/C-RNA region avoids interruptions of main pgRNA splicing variants and truncated 3′ terminals. To accurately detect blood HBV RNA, the amplification site needs to be selected carefully. Research also showed that treatment-naive individuals had high proportions of spliced pgRNAs and 3′ terminal-truncated pgRNAs, despite an absence of influence of HBeAg status. In early stages of treatment, these proportions declined in patients receiving nucleoside analogs (NAs), but climbed once again after 2 years of treatment. Based on these findings, it appears that long-term treatment with NAs exacerbates the interference induced by splicing variations and truncated 3′ terminals of pgRNA. This highlights the necessity of appropriate amplification region selection, particularly in scenarios involving long-term treatment.

## 4. Implications for blood product safety screening

Serum HBV RNA detection holds great potential as a biomarker for blood product safety screening. The presence of HBV RNA in blood units indicates active viral replication and the potential risk of transmission. By implementing sensitive and standardized assays for serum HBV RNA detection, blood banks and transfusion services can enhance their screening protocols, ensuring the exclusion of HBV-infected units and minimizing the risk of transmission to recipients (15).

Currently, the detection of HBV DNA is the primary method utilized for checking the safety of blood products. Nevertheless, the shortcomings of HBV DNA measurement, such as the disappearance of detectable DNA after antiviral treatment, call for the investigation of other biomarkers. The permanence of serum HBV RNA and the fact that it directly reflects the activity of the virus make it a desirable supplement to the screening procedures that are already in use. According to Kramvis's work, the incorporation of serum HBV RNA detection into blood product screening processes has the potential to considerably increase the accuracy and efficiency of identifying HBV-infected blood units, thereby protecting the health of recipients and ensuring the safety of the transfusion process (16).

## 5. Future directions and conclusion

In chronic HBV infection, the identification and validation of serum HBV RNA as a biomarker have opened up new pathways for improved diagnostic efficiency and blood product safety screening. To fully investigate its clinical value and establish standardized techniques for its detection, additional research and validation studies are required. To validate the findings and evaluate the generalizability of the optimized tests, further studies should be conducted on CHB patients comprising larger patient cohorts. In addition, research should be conducted into the underlying processes of lower reverse transcription efficiency of pgRNA splicing variants in order to achieve a full understanding of the effect that these variants have on the amount of HBV RNA found in serum. The interpretation of serum HBV RNA levels could be improved further by gaining a better understanding of the trans-packaging mechanism of pgRNA and the implications this has on the efficiency of reverse transcription.

In conclusion, the detection of HBV RNA in serum represents a substantial development in the effectiveness of the diagnostic process for chronic HBV infection. Recent research has shown that splicing variations and 3′ terminal truncations can have an effect on the amount of HBV RNA that can be measured in serum. These findings highlight how important it is to optimize assays and choose optimal amplification locations. The accuracy and sensitivity of HBV RNA quantification can be enhanced by healthcare providers by using standardized and sensitive assays. This enables improved monitoring of viral activity, assessment of treatment response, and informed decision-making regarding treatment discontinuation. In addition, the detection of HBV RNA in serum demonstrates excellent potential as a biomarker for the evaluation of the safety of blood products. Its incorporation into screening processes has the potential to assist in the detection of HBV-infected blood units, hence maximizing the efficacy of blood transfusions and lowering the probability of disease transmission. To further investigate the clinical value of serum HBV RNA detection and its application in bigger cohorts of CHB patients and blood product screening programs, continued research and validation studies are necessary. These studies must be carried out. In the end, the incorporation of serum HBV RNA detection into clinical practice and the implementation of blood safety precautions helps to a more effective management of chronic HBV infection and moves the aim of HBV eradication closer to realization.

## Author contributions

SY and YG conducted the literature analysis and drafted the original manuscript. CZ revised and edited the manuscript. All authors approved the final version of manuscript.
